# Case report: Importance of early and continuous tocilizumab therapy in nephrotic syndrome associated with idiopathic multicentric Castleman disease: A case series

**DOI:** 10.3389/fmed.2022.1037032

**Published:** 2023-01-09

**Authors:** Daiki Kojima, Shintaro Yamaguchi, Akinori Hashiguchi, Kaori Hayashi, Kiyotaka Uchiyama, Norifumi Yoshimoto, Keika Adachi, Takashin Nakayama, Ken Nishioka, Takaya Tajima, Kohkichi Morimoto, Jun Yoshino, Tadashi Yoshida, Toshiaki Monkawa, Takeshi Kanda, Hiroshi Itoh

**Affiliations:** ^1^Division of Endocrinology, Metabolism, and Nephrology, Department of Internal Medicine, Keio University School of Medicine, Tokyo, Japan; ^2^Department of Pathology, Keio University School of Medicine, Tokyo, Japan; ^3^Apheresis and Dialysis Center, Keio University School of Medicine, Tokyo, Japan; ^4^Medical Education Center, Keio University School of Medicine, Tokyo, Japan

**Keywords:** idiopathic multicentric Castleman disease, renal pathology, secondary nephrotic syndrome, IL-6 inhibitor, tocilizumab, acute kidney injury, renal replacement therapy

## Abstract

Idiopathic multicentric Castleman disease (iMCD) is a systemic and polyclonal lymphoproliferative disease involving multiple organs, including the kidneys, due to the overproduction of interleukin-6 (IL-6). Recently, several reports have suggested that excessive IL-6 actions in iMCD could have a causal relationship with the development of diverse histopathological renal manifestations that cause nephrotic syndrome. However, the treatment for such cases remains unclear. We report a series of three cases of nephrotic syndrome due to iMCD that helps to delineate the importance of early and continuous therapy with the anti-interleukin-6 receptor antibody tocilizumab. First, treatment was suspended for infectious control, and the patient presented with nephrotic syndrome due to diffuse mesangial and endocapillary hypercellularity without immune deposits complicating acute kidney injury. Second, iMCD was treated with prednisolone alone. The patient suddenly developed nephrotic syndrome due to immune-complex glomerulonephritis, not otherwise specified, complicated with acute kidney injury. In the third case, nephrotic syndrome secondary to membranous glomerulonephritis was diagnosed, with a skin rash and IgE antibodies to tocilizumab, and was therefore treated with prednisolone alone. In contrast to the first two cases, the third progressed to end-stage renal disease on hemodialysis. Taken together, this series suggests that clinicians should maintain clinical vigilance for iMCD as a possible underlying component of nephrotic syndrome, since iMCD presents with a variety of renal pathologies. Prompt initiation and continuous administration of tocilizumab are likely key determinants of renal outcomes in such cases. In particular, when tocilizumab is suspended due to infection or in the perioperative period, consideration of its expeditious resumption should be made, taking into account both the withdrawal period and systemic conditions.

## 1. Introduction

Castleman’s disease (CD) is a polyclonal, non-neoplastic, lymphoproliferative disease first described in the 1950s by Benjamin Castleman (1, 2). From a clinical perspective, the CD can be identified by the distribution of affected lesions. One type of CD is unicentric CD (UCD), in which enlarged lymph nodes are confined to a single area, and multicentric CD (MCD), in which swollen lymph nodes are present in multiple areas (3). MCD is further classified by the presence of human herpesvirus-8 (HHV-8), and HHV-8-negative is defined as idiopathic MCD (iMCD) (4). MCD presents with hepatosplenomegaly, fever, malaise, sweating, anemia, skin rash, edema, pleural effusion, renal involvement, interstitial lung lesions, and arthralgia (3, 5). The onset of such systemic symptoms is acute to subacute in HHV-8-positive MCD but insidious in iMCD (6). Some iMCD cases share a group of clinical characteristics with TAFRO syndrome manifesting as thrombocytopenia (T), anasarca (A), fever (F), reticulin fibrosis (R), and organomegaly (O) (7, 8). TAFRO syndrome is often associated with endotheliopathy and adrenalitis (7–9). Notably, the TAFRO subtype of iMCD is defined as TAFRO syndrome with lymph node histopathology consistent with iMCD, and its aggressive clinical behavior includes renal insufficiency and high mortality, particularly in Asian men (8). Importantly, the pathogenesis is thought to be caused by overstimulation of the immune system by interleukin-6 (IL-6) produced in the enlarged lymph nodes (10). Therefore, international evidence-based consensus treatment guidelines for iMCD and TAFRO syndrome recommend glucocorticoid and IL-6 inhibitors (11, 12).

Renal manifestations, including nephrotic syndrome and end-stage renal disease (ESRD), are recognized as important complications of iMCD (11). However, their renal pathology remains unclear. For example, previous studies have shown that patients with iMCD present with diverse histopathological manifestations, including AA renal amyloidosis, IgA nephritis, and thrombotic microangiopathy (13–16). Given that transgenic mice overexpressing IL-6 demonstrated mesangial proliferative glomerulonephritis (17), and IL-6 inhibition by tocilizumab improved nephrotic syndrome due to membranous glomerulonephritis and renal amyloidosis in iMCD (18, 19), excessive IL-6 secretion likely plays an important role in the development of renal complications and systemic manifestations. However, the effects of initiation timing, suspension, or termination of the IL-6 inhibitor tocilizumab on renal outcomes in iMCD remain unknown. Herein, we report three cases of iMCD with nephrotic syndrome, demonstrating that prompt and continuous tocilizumab administration may be an important factor in the management of nephrotic syndrome and renal prognosis.

## 2. Case descriptions

### 2.1. Case 1

**FIGURE 1:**
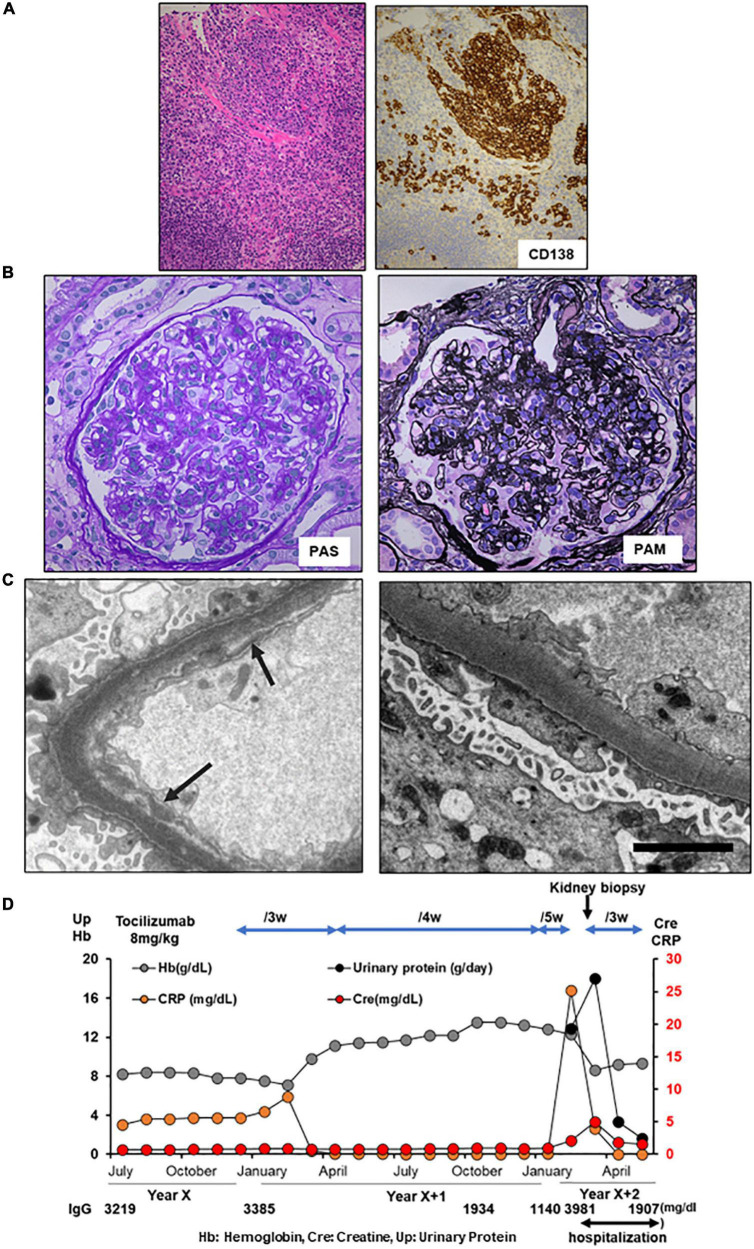
Histology and time course for Case 1. **(A)** Light microscopy of the external iliac lymph node exhibiting follicular hyperplasia (left lower) and infiltration by CD138^+^ plasma cells. **(B,C)** Renal biopsy. **(B)** Light micrograph showing diffuse mesangial and endocapillary hypercellularity. **(C)** Electron micrograph showing subendothelial widening with neo-densa (left, silver impregnation) and glomerular basement membrane thickening (right). Arrows indicate argyrophilic neo-densa. Scale bar, 2.5 μm. PAS: Periodic acid–Schiff. PAM: Periodic acid methenamine. **(D)** Clinical time course of Case 1. Hb, hemoglobin, Cre, creatine, Up, urinary protein.

A 53-year-old Japanese woman with iMCD and type 2 diabetes was admitted to our hospital for the treatment of cellulitis and acute kidney injury (AKI). At the age of 51 years, the patient had polyclonal hypergammaglobulinemia (IgG, 3,219 mg/dl; IgA, 659 mg/dl; IgM, 151 mg/dl), elevated levels of IL-6 (38.5 pg/ml) and soluble interleukin-2 receptor (912 U/mL), anemia (Hb 8.5 g/dl) (Table 1), and multiple enlarged lymph nodes. She underwent a lymph node biopsy in the right external iliac region and was diagnosed with iMCD (Figure 1A). Because the patient had type 2 diabetes mellitus on insulin therapy, she initially received 8 mg/kg of tocilizumab alone every 3 weeks, which was later decreased to every 5 weeks. On admission, her right lower leg appeared swollen, red, painful, and warm. She was diagnosed with cellulitis, and tocilizumab was discontinued for infection control. Her serum creatinine and 24-h urinary protein levels increased to 5.0 mg/dl and 18.1 g/day, respectively, with baselines of 0.9 mg/dl and 0.6 g/day, respectively. The selectivity index was 0.48. Inflammatory markers including peak C-reactive protein (CRP) level, D-dimer, and ferritin were 25.19 mg/dl, 30.2 μg/ml, and 193 ng/ml, respectively (Table 1). Renal biopsy identified 10 glomeruli, of which five were globally sclerosed. The remaining glomeruli showed diffuse mesangial hypercellularity, with focal endocapillary hypercellularity (Figure 1B). Congo red staining results were negative. Immunofluorescence microscopy revealed no remarkable staining of the glomeruli. Electron microscopy demonstrated subendothelial widening with neo-densa and thickening of the glomerular basement membrane partly due to diabetes (Figure 1C). These features of glomerular injury are considered to represent endothelial injury, which is likely associated with iMCD. Therefore, tocilizumab (8 mg/kg) was resumed, leading to significant improvements in CRP levels, proteinuria, and kidney function. She was discharged after complete healing of cellulitis. Two months later, her serum creatinine and 24-h urinary protein levels had declined to 1.54 mg/dl and 1.6 g/day, respectively (Figure 1D).

**TABLE 1:** Clinical markers of the cases.

	Case 1	Case 2	Case 3	Normal range
Sex	Female	Male	Male	
Age (years old)	53	60	58	
Organ involvement	Kidney	Kidney	Kidney	
Urinary protein (g/24 h)	18.1	15.7	3.5	
Serum Creatinine (mg/dL)	5	1.92	2	(0.65–1.07)
IL-6 (pg/ml)	38.5	13.5	639	(<7)
Peak C-reactive protein (mg/dL)	25.19	2.67	9.48	(0–0.14)
Immunoglobulin G (mg/dL)	3,219	3,201	8,039	(870–1,700)
Albumin (g/dL)	2.2	2.1	2.4	(4.1–5.1)
Hemoglobin (g/dl)	8.5	6.3	10.3	(13.7–16.8)
Platelets (/μL)	28.4 × 10^4^	19.9 × 10^4^	28.7 × 10^4^	(15.8–34.8)
Ferritin (ng/mL)	193	298	117	(8–129)
D-dimer (μg/mL)	30.2	9	0.5	(0–1)
Soluble interleukin-2 receptor (U/mL)	912	1,195	2,043	(142–500)

### 2.2. Case 2

**FIGURE 2:**
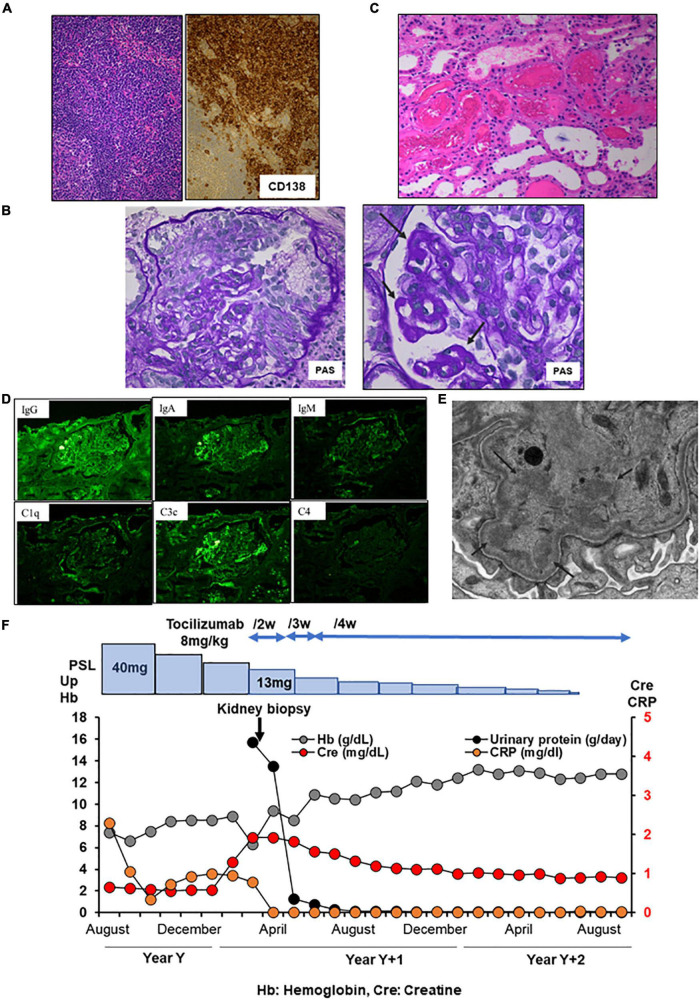
Histology and time course for Case 2. **(A)** Light micrograph of the lymph node showing follicular hyperplasia (left lower) and infiltration by plasma cells which are positive for CD 138. **(B–E)** Renal biopsy. **(B)** Light micrograph showing focal endo- and extra-capillary hypercellularity with hyaline deposits (arrows). **(C)** Tubular injury with red blood cell casts is observed. **(D)** Immunofluorescence micrograph showing granular mesangial and capillary staining for IgG, IgA, IgM, C1q, and C3. **(E)** Electron micrograph showing mesangial deposits (arrows). PAS: Periodic acid–Schiff. PAM: Periodic acid methenamine. **(F)** Clinical time course of Case 2. Hb, hemoglobin; Cre, creatine; Up, urinary protein.

A 60-year-old Japanese man with iMCD was admitted to our hospital with nephrotic proteinuria, reduced renal function, and anemia. At the age of 59 years, he presented with multiple enlarged lymph nodes. A biopsy of the right axilla revealed plasma cell infiltration, leading to a diagnosis of iMCD (Figure 2A). The patient had polyclonal hypergammaglobulinemia (IgG, 3,201 mg/dl; IgA, 487 mg/dl; IgM, 139 mg/dl), and high levels of IL-6 (13.5 pg/ml) and soluble interleukin-2 receptor (1,195 U/mL). In addition, peak CRP level was 2.67 mg/dl (Table 1). He was administered 40 mg of oral prednisolone daily, which was tapered. At the age of 60 years, he noticed progressive pretibial edema and an increase in body weight of 9 kg over 2 months. Laboratory investigations indicated AKI with a serum creatinine of 1.92 mg/dl and anemia with a hemoglobin of 6.3 g/dl. His 24-h urinary protein was 15.7 g/day and serum D-dimer was 9.0 μg/ml (Table 1). Renal biopsy revealed focal endo- and extra-capillary hypercellularity with hyaline deposits (Figure 2B). Tubular injury with red blood cell casts was also observed (Figure 2C). Immunofluorescence microscopy revealed 1 + to 2 + granular mesangial and capillary staining for IgG, IgA, IgM, C1q, and C3, corresponding to hyaline deposits (Figure 2D). Although large hyaline deposits were not observed by electron microscopy, small mesangial deposits were identified (Figure 2E). Systemic lupus nephritis was ruled out because we detected no apparent clinical manifestations of lupus, such as oral ulcers, subacute cutaneous or discoid lupus, and joint pain, and found a normal ds-DNA level (5.8 IU/ml) with a negative antinuclear antibody test and normal complement levels (C3 component 77 mg/dL, C4 component 21 mg/dL, respectively). In addition, monoclonal gammopathy was excluded based on a normal kappa/lambda ratio (0.89) and serum immunofixation electrophoresis. The patient was diagnosed with immune-complex glomerulonephritis, not otherwise specified, associated with iMCD. He was started on tocilizumab (8 mg/kg) every 2 weeks, plus 10 mg of prednisolone daily. Serum creatinine, CRP, and urinary protein excretion levels improved immediately. His 24-h urinary protein was 0.3 g/day at discharge. The patient remained in remission (Figure 2F).

### 2.3. Case 3

**FIGURE 3:**
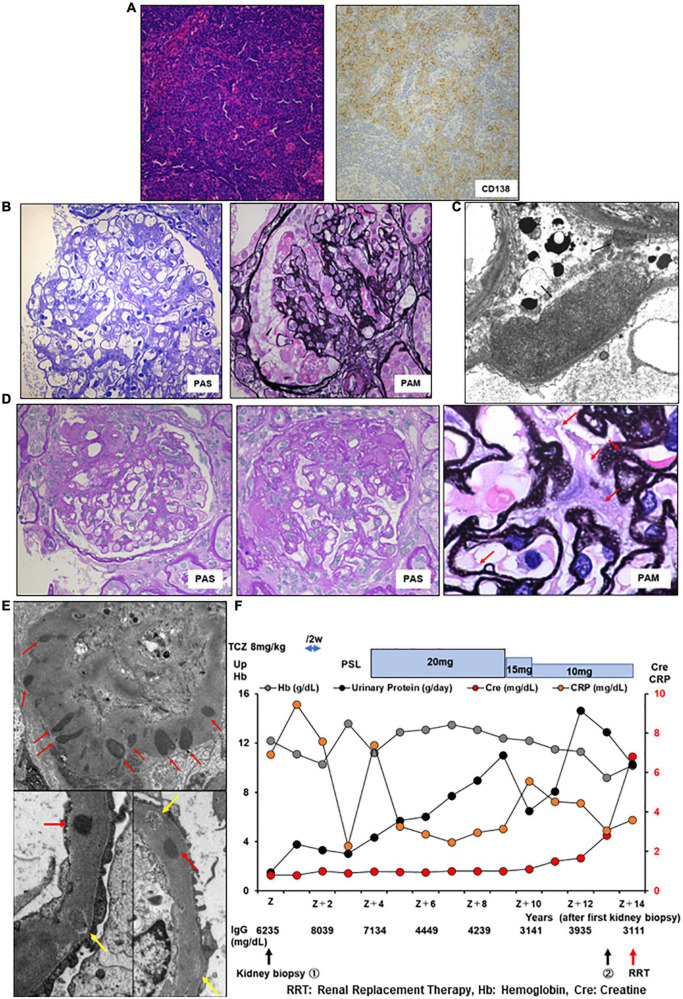
Histology and time course for Case 3. **(A)** Light micrograph of the lymph node showing follicular hyperplasia (left lower) and infiltration by plasma cells which are positive for CD 138. **(B,C)** First renal biopsy. **(B)** Light micrograph showing slight mesangial expansion and focal segmental glomerular sclerosis. **(C)** Electron micrograph showing focal subepithelial deposits (arrows). **(D,E)** Second renal biopsy. **(D)** Light micrograph showing focal global and segmental sclerosis with diffuse glomerular basement membrane lucencies (red arrows). **(E)** Electron micrograph showing subepithelial and intramembranous deposits (red arrows). Some of the intramembranous deposits are electron-lucent and granular appearance (yellow arrows). PAS: Periodic acid–Schiff. PAM: Periodic acid methenamine. **(F)** Clinical time course of Case 3. Hb, hemoglobin; Cre, creatine; Up, urinary protein; RRT, renal replacement therapy.

A 58-year-old Japanese man with iMCD and ESRD with nephrotic proteinuria was hospitalized for hemodialysis. At the age of 44 years, he presented with multiple enlarged lymph nodes, and a left axillary biopsy revealed iMCD (Figure 3A). His 24-h urinary protein was 1.5 g/day. Renal biopsy revealed a slight mesangial expansion and focal segmental glomerular sclerosis with podocyte hyperplasia (Figure 3B). Electron microscopy revealed only a few subepithelial deposits, which suggested atypical membranous glomerulonephritis (Figure 3C). The patient was monitored without therapy because he was asymptomatic. At the age of 46 years, laboratory investigations showed hemoglobin 10.3 g/dL, CRP 9.48 mg/L, polyclonal hypergammaglobulinemia (IgG, 8,039 mg/dl; IgA, 454 mg/dl; IgM, 146 mg/dl), IL-6 639 pg/ml, and 24-h urinary protein 3.5 g/day (Table 1). Although tocilizumab was initiated, the skin rash appeared soon after treatment initiation, and IgE antibodies to tocilizumab were detected. Therefore, treatment was stopped, and prednisolone monotherapy was initiated. However, the CRP level remained slightly elevated at approximately 2–3 mg/dl. As the degree of proteinuria increased, renal function gradually deteriorated. We suggested a rechallenge with tocilizumab; however, the patient did not consent. At the age of 57 years, 24-h urinary protein levels further increased to 6.948 g/day. Although the selectivity index was 0.36, the patient underwent a second renal biopsy. Light microscopy revealed focal global and segmental sclerosis with diffuse glomerular basement membrane lucency on sliver staining (Figure 3D). Congo red staining results were negative. Electron microscopy revealed the presence of subepithelial and intramembranous deposits (Figure 3E). The patient was diagnosed with membranous glomerulonephritis associated with iMCD. His renal function further diminished, and he required renal replacement therapy (Figure 3F).

## 3. Discussion

Our case series demonstrated that (1) iMCD should be acknowledged as an underlying cause of nephrotic syndrome, albeit rare, and (2) immediate and uninterrupted tocilizumab therapy is likely essential for the management of iMCD-associated renal complications, especially nephrotic syndrome. In addition, our cases with hypergammaglobulinemia without thrombocytopenia, anasarca, or organomegaly could be categorized as non-TAFRO iMCD (20).

Many cases of CD are associated with renal dysfunction (21–23) and MCD is clinically characterized by renal dysfunction (24). However, renal pathology in MCD is diverse; a recent investigation of 64 patients and previous case reports revealed that the renal pathologies underlying renal derangements included amyloidosis, membranoproliferative glomerulonephritis, thrombotic microangiopathy, membranous glomerulonephritis, minimal change disease, and mesangial proliferative glomerulonephritis (18, 19, 25–28). IgA nephropathy (29) and interstitial nephritis (30, 31) have also been reported. Although renal function in MCD is reportedly an important prognostic factor for survival (32), MCD is not well recognized as a cause of renal dysfunction, especially secondary nephrotic syndrome, probably because of the highly diverse renal pathological findings.

Our three cases of iMCD presenting with nephrotic syndrome consistently displayed diverse renal pathologies: glomerular endothelial injury, immune-complex glomerulonephritis, and membranous glomerulonephritis. The mechanism(s) responsible for the multiple renal pathologies that cause nephrotic syndrome in iMCD remains unclear but may involve the overproduction of IL-6 in the affected lymph nodes (33). Interestingly, it was reported that high circulating levels of IL-6 might contribute to the glomerular loss of vascular endothelial growth factor (VEGF) expression in iMCD (34), thereby triggering renal thrombotic microangiopathy (33, 35), as in Case 1. Podocyte-specific *Vegf*-knockout mice exhibit glomerular endothelial damage (36, 37). In addition, high levels of IL-6 are known to promote B cell activation, which in turn induces glomerulonephritis with immune complex (22), as in Case 2.

As the overproduction of IL-6 could be involved in the development of renal complications in iMCD, it has been reported that IL-6 inhibitors are effective for prevention and treatment of iMCD-associated nephrotic syndrome (18, 19, 38, 39). Although a previous report showed that nephrotic syndrome in iMCD was relieved by steroids alone (40), international, evidence-based, consensus treatment guidelines for iMCD recommend treatment with IL-6 inhibitors siltuximab or tocilizumab, regardless of disease severity. For severe iMCD, based on the Castleman Disease Collaborative Network severity classification, IL-6 inhibitor plus glucocorticoid is recommended (11). As we experienced in Case 2, given that a possible causal relationship between excessive IL-6 production and renal complications, including nephrotic syndrome, is established, we strongly recommend introducing tocilizumab from the induction phase of treatment for iMCD.

In contrast, over the course of iMCD treatment, clinicians are required to suspend or discontinue tocilizumab because of side effects such as infection and allergic reactions, as in cases 1 and 3, respectively. Although lifelong administration of tocilizumab is important for control (41, 42), infection is a crucial complication of IL-6 inhibitors; 7% of patients with MCD receiving tocilizumab develop cellulitis (34). A previous case report revealed that an 8-week temporary cessation after long-term administration of tocilizumab due to orthopedic surgery led to a relapse of iMCD (42). Importantly, 1 μg/mL of serum-free tocilizumab, which can be maintained over 4 weeks after a single administration of 8 mg/kg tocilizumab, is the lowest concentration required to block IL-6 signaling (43, 44). Indeed, our Case 1 demonstrated that a 6-week suspension of tocilizumab due to infection caused nephrotic syndrome. Taken together, we speculate that tocilizumab should be carefully reintroduced within 4 weeks based on the severity of the infection.

Case 3 also illustrates the important role of tocilizumab in protecting renal function in iMCD. The patient was allergic to tocilizumab and had been receiving prednisolone monotherapy for approximately 12 years. A case report showed that nephrotic syndrome in iMCD was successfully resolved after corticosteroid monotherapy (40). However, our patient was in the nephrotic range of proteinuria due to secondary membranous glomerulonephritis, with a CRP level of approximately 3 mg/dl, ultimately resulting in ESRD requiring hemodialysis. Importantly, a recent case report demonstrated that an Asian male with the TAFRO subtype of iMCD required hemodialysis twice and could withdraw from dialysis by initiating IL-6 blockers (45). To the best of our knowledge, this is the first case report of chronic maintenance renal replacement therapy for iMCD. Together, these results demonstrate that tocilizumab may be a key determinant of renal prognosis in iMCD, especially in patients with nephrotic syndrome.

Our case series demonstrates the importance of recognizing iMCD as a possible causative disease of secondary nephrotic syndrome with various histopathological findings, which could result in ESRD on dialysis, albeit rarely. Our cases also support the contention that early and continuous administration of tocilizumab may not only be effective in controlling iMCD disease activity but also in preventing the onset and progression of associated renal complications, such as nephrotic syndrome. Specifically, when tocilizumab should be suspended due to infection or the perioperative period, expediting the resumption of tocilizumab is required, taking into account its withdrawal period as well as systemic conditions.

## Data availability statement

The original contributions presented in this study are included in the article/supplementary material, further inquiries can be directed to the corresponding author.

## Ethics statement

Informed consent was obtained from the individual(s) for the publication of any potentially identifiable images or data included in this article.

## Author contributions

DK and SY wrote the manuscript. All authors took clinical care of the patient and have read and approved the final manuscript.
